# A new *in vivo* model of pantothenate kinase-associated neurodegeneration reveals a surprising role for transcriptional regulation in pathogenesis

**DOI:** 10.3389/fncel.2013.00146

**Published:** 2013-09-09

**Authors:** Varun Pandey, Hagit Turm, Uriya Bekenstein, Sagiv Shifman, Sebastian Kadener

**Affiliations:** ^1^Biological Chemistry Department, Silberman Institute of Life Sciences, The Hebrew University of JerusalemJerusalem, Israel; ^2^Department of Genetics, Silberman Institute of Life Sciences, The Hebrew University of JerusalemJerusalem, Israel

**Keywords:** NBIA, PKAN, PanK, circadian, CoA, *Drosophila*

## Abstract

Pantothenate Kinase-Associated Neurodegeneration (PKAN) is a neurodegenerative disorder with a poorly understood molecular mechanism. It is caused by mutations in Pantothenate Kinase, the first enzyme in the Coenzyme A (CoA) biosynthetic pathway. Here, we developed a *Drosophila* model of PKAN (tim-fbl flies) that allows us to continuously monitor the modeled disease in the brain. In tim-fbl flies, downregulation of *fumble*, the *Drosophila PanK* homologue in the cells containing a circadian clock results in characteristic features of PKAN such as developmental lethality, hypersensitivity to oxidative stress, and diminished life span. Despite quasi-normal circadian transcriptional rhythms, tim-fbl flies display brain-specific aberrant circadian locomotor rhythms, and a unique transcriptional signature. Comparison with expression data from flies exposed to paraquat demonstrates that, as previously suggested, pathways others than oxidative stress are affected by PANK downregulation. Surprisingly we found a significant decrease in the expression of key components of the photoreceptor recycling pathways, which could lead to retinal degeneration, a hallmark of PKAN. Importantly, these defects are not accompanied by changes in structural components in eye genes suggesting that changes in gene expression in the eye precede and may cause the retinal degeneration. Indeed tim-fbl flies have diminished response to light transitions, and their altered day/night patterns of activity demonstrates defects in light perception. This suggest that retinal lesions are not solely due to oxidative stress and demonstrates a role for the transcriptional response to CoA deficiency underlying the defects observed in *dPanK* deficient flies. Moreover, in the present study we developed a new fly model that can be applied to other diseases and that allows the assessment of neurodegeneration in the brains of living flies.

## Introduction

PanK-associated neurodegeneration (PKAN, NBIA1, HSS, OMIM 234200) is a monogenic neurodegenerative motor-disorder that results from diverse mutations of the human *PanK2* gene (Zhou et al., 2001). In PKAN, symptoms begin in childhood and progressively worsen resulting in a drastically reduced life span (Gregory et al., 2009) as well as retinal degeneration, a hallmark of the disease (Egan et al., 2005). PKAN patients present a specific MRI pattern, representing focal iron accumulation in the *globus pallidus* and *substantia nigra*, despite overall normal levels of iron in the whole brain (Gregory and Hayflick, 2005). The iron accumulation correlates with neural damage and mitochondrial lesions; however, the etiological link between *PanK2*-loss and the neurodegenerative phenotype is not well understood. In addition to iron accumulation, some PKAN patients have tau-positive neurofibrillary tangles and Lewy bodies in cortical and subcortical brain regions (Neumann et al., 2000).

Pantothenate kinase enzymes (PanK) catalyze the phosphorylation of pantothenate, better known as vitamin B5, in the first and rate-limiting step of the coenzyme-A biosynthetic pathway (Robishaw and Neely, 1985). Coenzyme A (CoA) is an essential metabolic cofactor that plays a central role in numerous biological processes including the tricarboxylic acid cycle and the oxidation of fatty acids (Vagelos, 1964). The *PanK2* gene is one of four *PanK* genes in mammals, and diverse loss-of-function mutations in this gene in humans result in PKAN (Gregory and Hayflick, 2005; Kotzbauer et al., 2005; Leonardi et al., 2007). A *PanK2*-knock-out (KO) mouse was developed in 2005, yet it recapitulated only few features of the disease like retinal degeneration (Kuo et al., 2005) and impaired mitochondrial function (Brunetti et al., 2012). The mutant mice did not suffer from movement disorders nor did these mice show signs of neurodegeneration, implying that the other *PanK* genes may compensate for *PanK2* loss in mice (Leonardi et al., 2007).

Recent work with *Drosophila* models has provided important insights into the cellular lesions that play roles in PKAN pathology. A *Drosophila* hypomorphic mutation in *fumble*, the *Drosophila PanK2* homologue (*dPanK*), results in flies with severe motor impairment (Afshar et al., 2001; Yang et al., 2005). *Fumble* flies have brain lesions and defective neurological functions (i.e., paralysis and impaired climbing ability) (Wu et al., 2009). These strong phenotypes are rescued by expression of the human *PanK2*, underscoring the strong functional similarity between *Drosophila* and human *PanK* genes (Wu et al., 2009).

Initial work suggested an important role for oxidative stress in PKAN pathogenesis. More specifically reduction of CoA level facilitates increased protein oxidation, and mitochondrial dysfunction, which deteriorate intra mitochondrial homeostasis (Rana et al., 2010; Siudeja et al., 2011; Brunetti et al., 2012). However, it is clear that pathways other than oxidative stress are key contributors to PKAN pathogenesis.

For example, the low levels of CoA led to impaired histone and tubulin acetylation in both the *Drosophila* model and mammalian cells in culture, which may provoke many downstream effects. In addition, mitochondrial dysfunction in *fumble* mutants may be linked to their reported hypersensitivity to oxidative insults (Wu et al., 2009; Rana et al., 2010). Because the *fumble* flies are severely sick and survive only a few days after eclosion (Afshar et al., 2001; Yang et al., 2005; Bosveld et al., 2008), their use for studying neuro-specific aspects of the disease and for genetic screens is precluded. New disease models that allow neuron-specific features of the disease to be evaluated in a simple manner and that allow assessment of neuronal dysfunction rather than neuronal death are needed.

Retinal degeneration is one of the landmarks of PKAN and is observed in both fly (Yang et al., 2005; Wu et al., 2009) and mouse models of *dPanK* deficiency (Kuo et al., 2005). It is believed to be caused by downstream effect of CoA deficiency, mitochondrial dysfunction and oxidative stress (Koeppen and Dickson, 2001; Gregory and Hayflick, 2005). Recently, another pathway that can lead to retinal degeneration was found. More specifically, it was demonstrated that mutations in the *pdh* gene lead to a light-dependent loss of rhodopsin and retinal degeneration (Wang et al., 2010). The requirement of *pdh* for eye integrity is related to the recently discovered requirement for an enzymatic visual cycle in *Drosophila*. This visual cycle is necessary for chromophore recycling after the release of the light-activated rhodopsin and in *Drosophila* is directed by *pdh* (Wang et al., 2010). No connection between this pathway and the retinal degeneration observed in PKAN models and patients has been ever established.

In the present study, we developed a new type of PKAN model in which the neuronal nature of the disease can be studied in living flies. We chose to suppress *dPanK* in circadian clock cells as these cells drive a well-defined and easy to measure behavior (daily rhythms in locomotor activity). Flies, like most animals, display rhythmic patterns of activity that are the consequence of internal timing mechanism: the circadian clock (for review see Hall, 2003). Circadian clocks work in a cell-autonomous basis and are based on complex transcriptional feedback loops. Although many cells in the fly body display transcriptional/molecular oscillations, circadian rhythms in locomotor activity are generated by only 75–100 neurons in the fly brain (Hall, 2003). Our PKAN-model flies (tim-fbl) display many features of PKAN patients. In addition tim-fbl flies have disrupted circadian locomotor patterns and a unique transcriptional signature, which strongly suggests that many of the defects observed in PKAN pathogenesis may be related to a specific transcriptional signature rather than mere consequence of oxidative stress.

## Results

### Development of new drosophila models of PKAN

Due to the experimental limitations of the current fly PanK-deficiency model (*fumble* flies), we decided to generate new PKAN models. In order to do so, we utilized a publicly available UAS-RNAi transgene targeted against the endogenous *dPanK* (*fbl*) mRNA and the tim-gal4 driver to express it exclusively in circadian tissues. This combination of transgenes restricts the expression of the transgene (and hence the silencing of the endogenous *fbl* gene) to only tissues harboring a circadian clock. These tissues include the eyes, the fat body, and the circadian neuronal network. This model allows us to follow the disease by the use of specific markers that are related to circadian behavior and minimizes effects on the overall health of the model flies.

We generated two different RNAi models. The first one used the tim-gal4 driver in combination with a UAS-fbl RNAi transgene (herein referred to as tim-fbl flies). In the second, UAS-fbl RNAi is co-expressed with a UAS-Dcr2 transgene that has been shown to generally increase the strength of UAS-RNAi transgenes (tim-dcr2-fbl flies). As the tim-GAL4 driver is broadly expressed during development, it is possible that this silencing of *fumble* may result in a certain degree of embryonic or post embryonic lethality (as in the *fumble* hypomorphic strain). Indeed, tim-fbl and tim-dcr2-fbl flies display high pre-eclosion lethality (we observed 94.6 and 97% of developmental lethality for Tim-fbl and tim-dcr2-fbl flies). This is similar to what has been described for *fumble* mutants (Afshar et al., 2001).

**Figure 1:**
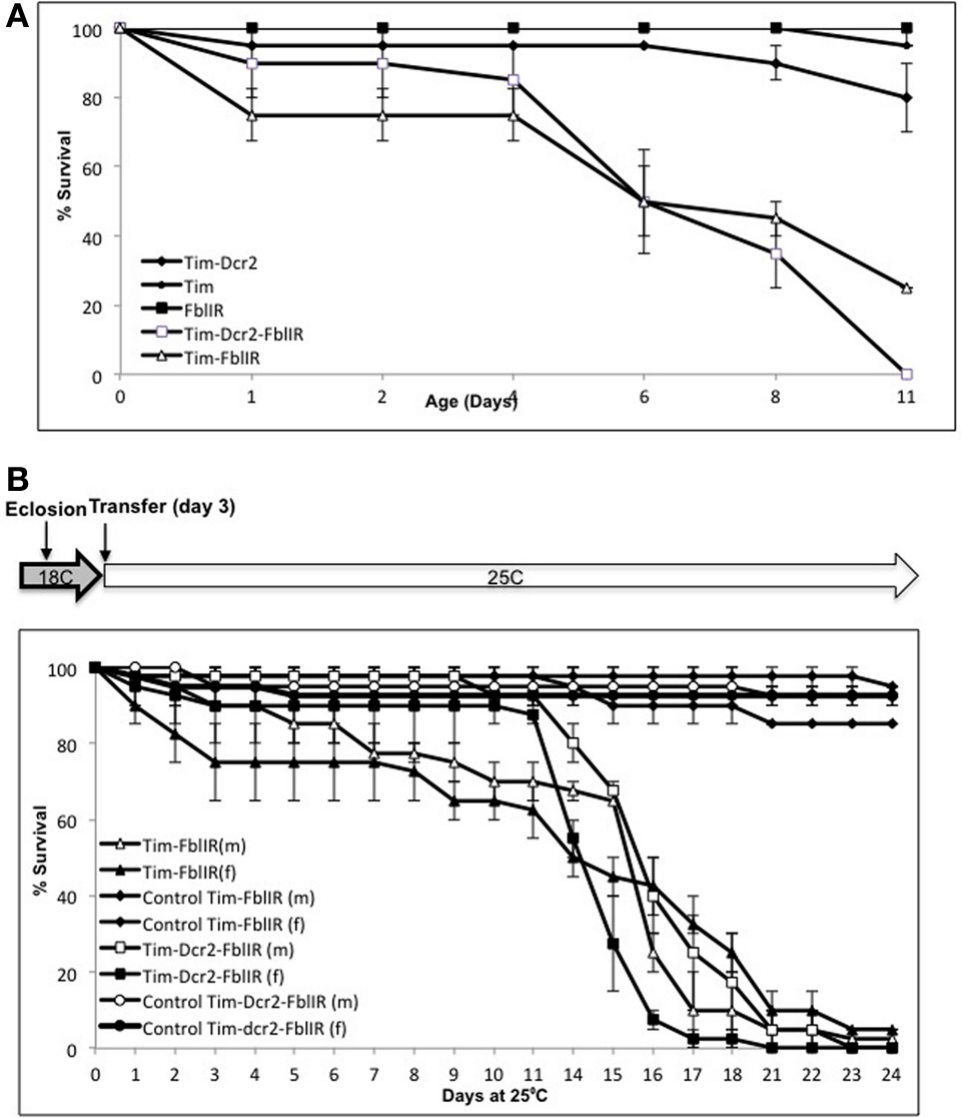
**A new cell-specific model of *dPanK* deficiency. (A)** Downregulation of *fumble* in *timeless*-expressing cells reduces life span. Cohorts of 20 flies per vial (*n* = 3) were grown in controlled conditions and counted; both models show a median life span of ~6 days, and curves of tim-fbl and tim-dcr2-fbl flies and their corresponding controls are significantly different from each other (Wilcoxon paired test; *P* < 0.001 for both). **(B)** When tim-fbl flies are raised at 18°C, the downregulation of *fumble* can be strengthen during adulthood. Both males and females flies raised at 18°C and transferred to 25°C 3 days after their eclosion have a median life span of ~15 days and curves of control vs. tim-fbl and tim-dcr2-fbl flies are significantly different from each other (Wilcoxon paired test; *P* < 0.001 for both).

In order to test whether our model recapitulates the short life times of PKAN patients and *fumble* mutants, we performed life-span assessment of control, tim-fbl, and tim-dcr2-fbl flies. Both tim-fbl and tim-dcr2-fbl flies display shorter life spans than wild-type flies with all PKAN flies dying within 2 weeks of life (Figure 1A). The diminished life span of tim-fbl flies may result from developmental defects, adult-related defects, or both. Because of the limited life span of these mutant flies, we utilized growing conditions that minimize the silencing of *fumble* during development. Briefly, we utilized a similar genetic approach but raised the tim-fbl or tim-dcr2-fbl flies at 18°C, a temperature at which the activity of the GAL4-UAS system is minimal (Duffy, 2002). In tim-fbl flies raised under these conditions, we observed minimal developmental lethality, suggesting that the expression of *fumble* RNAi is limited during development. In order to induce the expression of the UAS-RNAi transgene we transferred 3-day-old flies to 25°C, a temperature in which the GAL4-UAS system is more active. The tim-fbl flies were extremely sensitive to the expression of the UAS-dPanK RNAi transgene, and all flies died within 3 weeks (Figure 1B). Thus, increasing *fumble* knock-down during adulthood significantly shortens the life spans of tim-fbl and tim-dcr2-fbl flies. Importantly, we verified *fbl* downregulation by Real-Time PCR for both flies raised at 18 and 25°C (see below, Figure 6C and data not shown, see also Supplementary File 1).

### Tim-fbl flies exhibit increased sensitivity to oxidative stress

**Figure 2:**
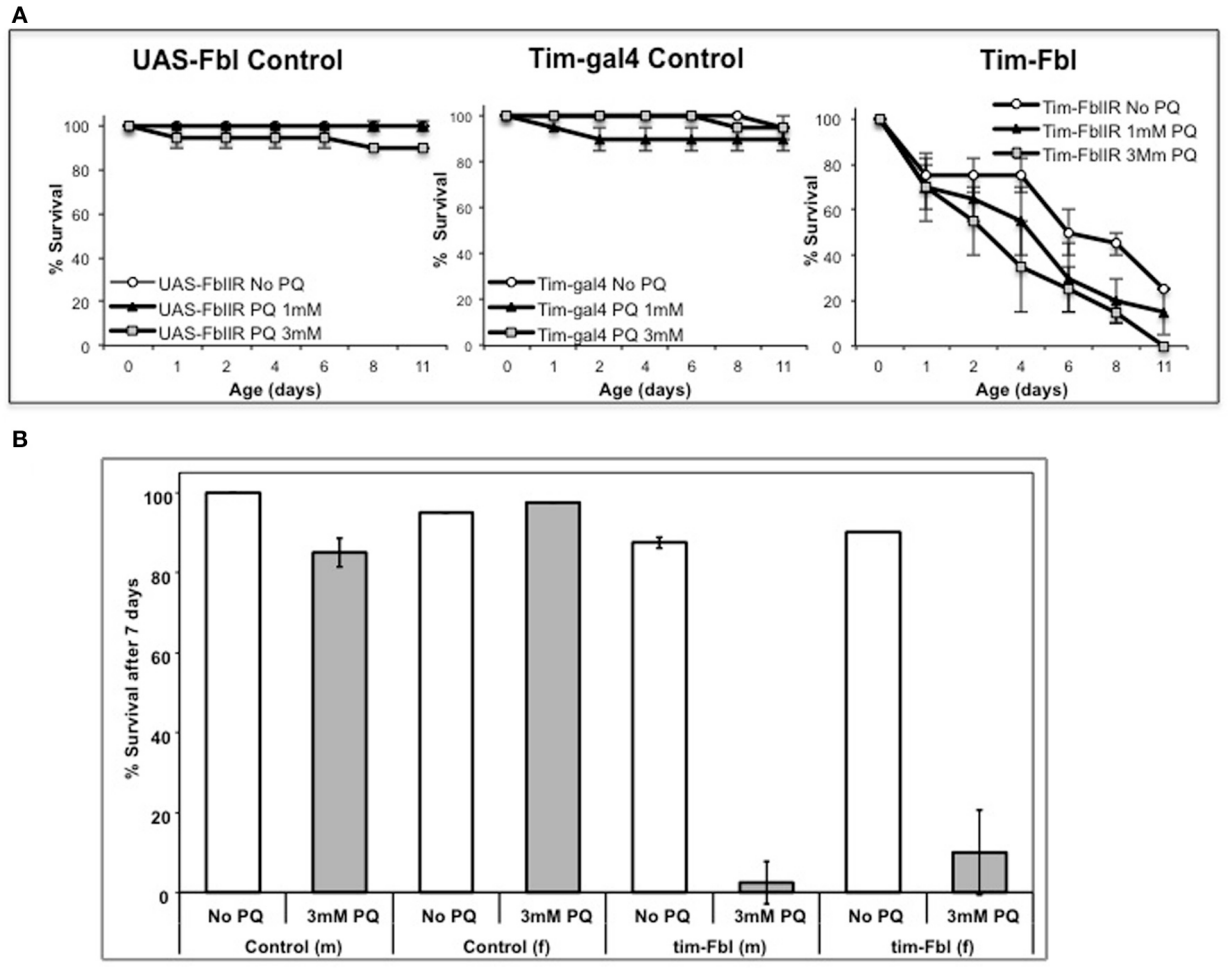
**Tim-fbl flies are hypersensitive to oxidative stress. (A)** Survival curves denote the effects of two different doses of paraquat on the life span of tim-fbl or wild-type flies. The tim-fbl flies and two types of genetic control flies (UAS-fbl IR and tim-gal4) were grown on paraquat-containing food. Paraquat feeding resulted in lethality to tim-fbl flies in a dose-dependent manner, but had little effect on life span of control flies. **(B)** A similar experimental was performed in tim-fbl flies initially raised at 18°C then transferred on day 3 after eclosion to food containing 3 mM paraquat and maintained in 25°C (20 flies per vial, *N* = 2). Ratio of flies surviving after 7 days of paraquat feeding was drastically changed in both male (m) and female (f) tim-fbl flies, but not in control flies carrying only the fbl-RNAi transgene.

One of the key features of PKAN is mitochondrial dysfunction that leads, at least in animal models, to hypersensitivity to oxidative stress (Wu et al., 2009; Rana et al., 2010). In order to establish tim-fbl flies as a reliable PKAN model, we determined the life span of tim-fbl and control flies in presence of the oxidative stress promoting-agent paraquat. We utilized a chronic paraquat exposure paradigm, in which after eclosion flies are given food containing this drug. Paraquat exposure has a strong and dose-dependent effect on the life span of tim-fbl but little effect on that of control flies (Figure 2A), further validating tim-fbl flies as a model for dPanK deficiency. In order to validate the temperature-inducible model, we grew tim-fbl or control flies at 18°C, transferred them to 25°C in presence or absence of 3 mM paraquat in the food source, and determined the fraction of surviving flies after 1 week. Paraquat exposure had little or no effect on life spans of male or female control flies. In contrast, less than 10% of the tim-fbl flies fed with paraquat survived for a week (Figure 2B), demonstrating that downregulating *dPanK* leads to oxidative stress susceptibility.

### Tim-fbl flies display brain-related circadian defects

In order to assess *in vivo* the activity of the circadian transcriptional cycle in tim-fbl flies, we utilized a *timeless-*luciferase (*tim-*luc) transgene, which is a validated system to assess circadian transcription (Allada et al., 2003) The expression of the *timeless* promoter (and hence the *tim-luciferase* transgene) is directed by the by the transcription factors CLOCK and CYCLE (CLK-CYC heterodimer). We generated tim-fbl flies that also carry a copy of a *tim-*luc transgene. We followed expression of the reporter by quantifying the amount of light emitted from these living flies due to the transgenic luciferase activity. We compared the emitted light measured over a week from tim-fbl and control flies that carry the same *tim*-luc transgene.

**Figure 3:**
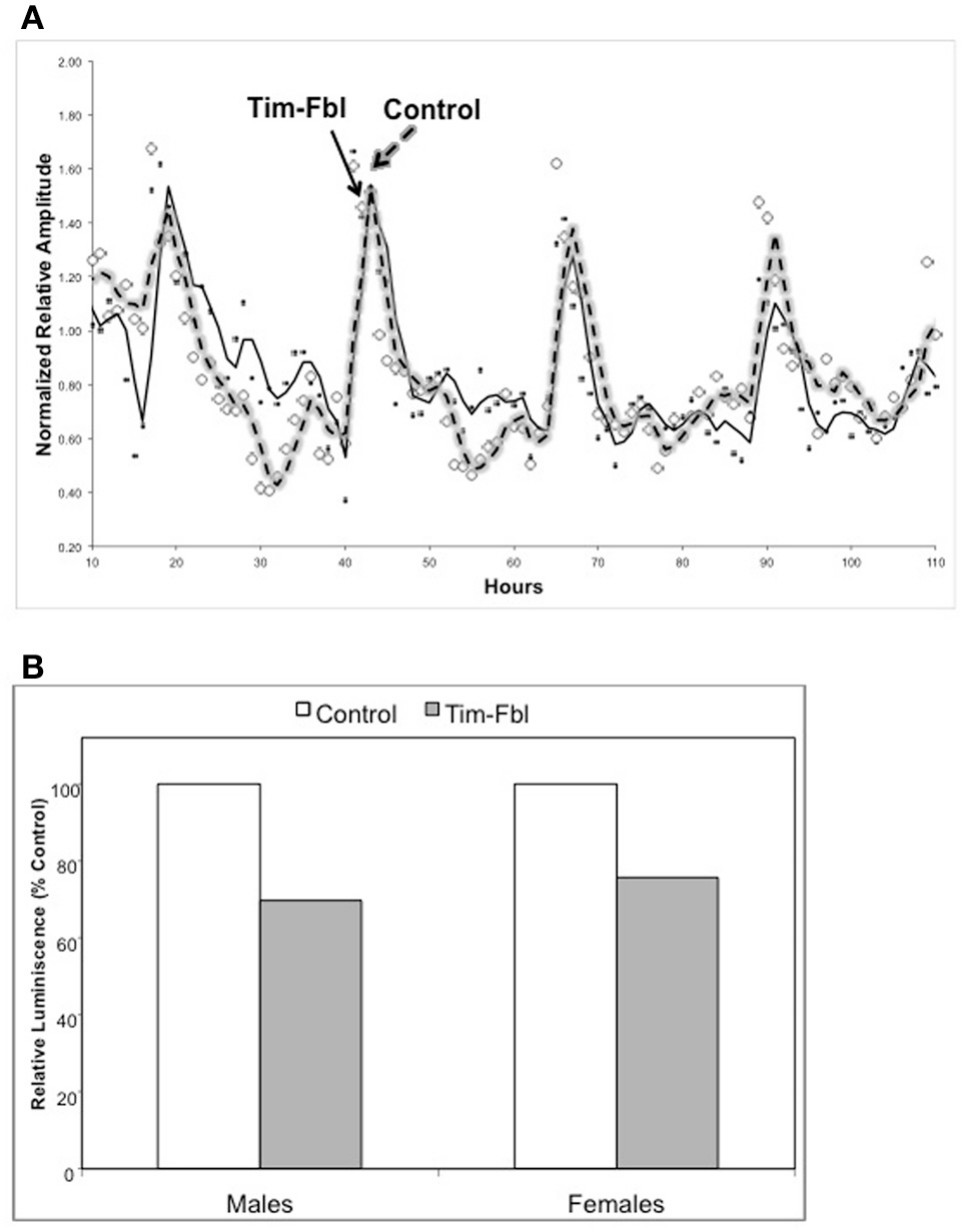
**The tim-fbl flies display normal circadian molecular rhythms but reduced luciferase levels. (A)** Male tim-fbl and control flies, both harboring a tim-luciferase transgene fed with Luciferine and maintained in a 12:12 light:dark conditions. Light emissions were measured from live flies. The emission pattern was not impaired in tim-fbl flies, either in relative amplitude of oscillation or in the oscillation period. **(B)** Overall emission was reduced by ~30% in tim-fbl males and females with respect to controls. (*n* = 16).

Both male and female tim-fbl flies showed temporal bioluminescence patterns similar in phase and amplitude to control flies (Figure 3A and data not shown). Therefore, the observed phenotype indicates that the general CLK-CYC driven transcription profile is normal in tim-fbl flies, consistent with current knowledge that does not link *fumble* expression to the circadian clock or to transcription in general. Nevertheless, tim-fbl flies displayed reduced levels of luciferase on average (reduced ~30% relative to control levels, Figure 3B), which could be due to lower ATP levels (see Discussion).

**Figure 4:**
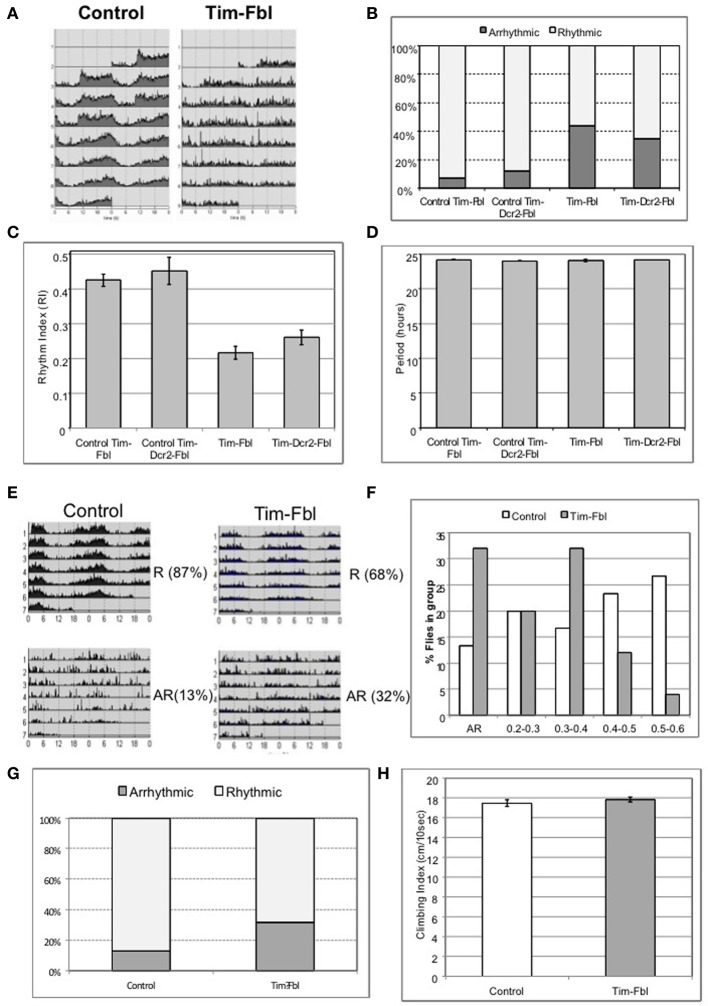
**The tim-fbl flies display altered circadian behavior. (A)** Locomotor behavior of tim-fbl flies is characterized by reduced rhythmicity in respect to controls. Actograms of female tim-fbl and controls represent activity in constant conditions immediately after 3 days of 12:12 light:dark entrainment and show reduced rhythmicity of tim-fbl flies. **(B)** Quantification of the rhythmicity of locomotion by the rhythm index (RI). **(C)** The mean Rhythm Index (RI) of tim-fbl and tim-dcr2 females is strongly reduced of that seen in control flies, showing dPANK downregulation in tim cells affects the specific and robust behavioral output of the tim-expressing neuronal network. **(D)** The period length of females from control and Tim-Fbl flies is similar suggesting that the molecular clock of the tim-expressing neuronal network of cells is not impaired. **(E)** Locomotor activity patterns of the inducible tim-fbl model. The tim-fbl flies were raised at 18°C and transferred to 25°C upon eclosion for measurement of locomotor activity. Control and tim-fbl Flies displaying rhythmic (RI > 0.2) or arrhythmic activity patterns were grouped, and their average profile plotted. The percentage indicates the percentage of flies in each group (R, rhythmic and AR, arrhythmic). **(F)** Tim-Fbl flies have weaker rhythms than control flies. Histogram representing relative frequency distribution of flies across rhythm indexes (RI) intervals. To construct this graph, we evaluated the RI of each control or Tim-Fbl fly and calculated the frequency of flies at each indicated RI interval (*n* = 64). **(G)** Quantification of the rhythmicity of locomotion by the rhythm index (RI) of control and tim-Fbl females raised at 18°C and transferred to 25°C. This quantification of the behavior showed in Figure 3D. **(H)** Young tim-fbl flies have intact climbing ability, suggesting intact health. Groups of 20 flies/group per genotype (*n* = 5) were tapped to bottom of graded cylinder and allowed to climb for 15 s and photographed. The distance climbed by individual flies showed no difference between young tim-fbl flies and controls.

The tim-fbl model developed in this study is based on the downregulation of *dPanK* in both neuronal and non-neuronal tissue. While we believe that the short life span of tim-fbl flies is likely consequence of *dPanK* deficiency in non-neuronal tissue, neuronal downregulation of this enzyme in circadian neurons should lead to defects in circadian rhythm in locomotor activity. We therefore, assayed the locomotor activity of tim-fbl and control flies. In all these tests we focused on females, as we postulated that given their less robust behavioral rhythms (Hall, 2003), reduction of *fumble* should result in more notable effects than in male flies. Indeed, whereas control flies have robust 24-h rhythms of activity, both tim-fbl and tim-dcr2-fbl female flies display aberrant circadian rhythms in their locomotor activity (Figures 4A,B). We measured these aberrant rhythms by determining the number of rhythmic flies (Figure 4B) or computing the average rhythm strength (Figure 4C). The robustness and persistence of the rhythms were strongly affected in tim-fbl flies, but the periodicity of the rhythms was not, supporting the idea that *fumble* downregulation does not have an effect on the molecular pacemaker (Figure 4D), which is a key determinant of the circadian period (Kadener et al., 2008). Given the relatively small defects on circadian transcription (Figure 3A), we deduce that these locomotor defects are likely related to the neuronal function of the circadian neurons rather than to general defects on the circadian timekeeping machinery in these cells. Due to the lack of enhancement of the phenotypes by *dcr2* co-expression, further experiments were performed with the tim-fbl flies.

We decided to follow by testing if the behavioral defects are also present in the model with more limited downregulation of *dPanK* during development. As with the tim-fbl flies with developmental expression, a significant number of flies grown at 18°C and transferred to 25°C upon eclosion showed arrhythmic behavior (Figure 4E) and weaker rhythms (lower Rhythm Index, RI) than control flies (Figures 4F,G).

A general concern regarding the use of the tim-gal4 driver to suppress *fbl* expression is that expression is organism wide, and, hence, circadian behavioral phenotypes in locomotion may arise due to a general defects in health. In order to rule out this possibility, we tested tim-fbl flies for climbing ability, an assay used as an indicator of general health (Bonilla-Ramirez et al., 2011). We performed this assay in newly emerged tim-fbl flies. Newly emerged tim-fbl flies had climbing scores very similar to those of controls (Figure 4H), suggesting that suppression of *fumble* expression in the circadian cells does not drastically compromise the overall health of the adult flies, at least at this early age.

### Tim-fbl flies display a unique transcriptional profile

PKAN is usually classified as a neurodegenerative disease with prominent iron accumulation and mitochondrial dysfunction (Gregory and Hayflick, 2005). It has been assumed that oxidative stress can contribute to the disease (Hayflick, 2006). The path connecting CoA deficiency with oxidative stress and neurodegeneration has been addressed (Bosveld et al., 2008; Wu et al., 2009; Rana et al., 2010; Brunetti et al., 2012; Campanella et al., 2012), but not fully established. In order to investigate the pathways altered in our PKAN model, we used oligonucleotide microarrays to compare the transcriptomes of tim-fbl and control flies. In order to avoid bias resulting from sex and time (circadian) effects, we collected RNA from three-day-old females entrained in 12:12 light:dark cycles at two different circadian time points. As expected, *fumble* (the endogenous *dPanK* gene) was among the most downregulated genes in tim-fbl flies (3.5 fold, *P-*value = 1.15 × 10^−4^, False discovery rate (FDR) *q*-value < 0.05; Supplementary File 2). It should be noted that *timeless* drives the expression in approximately 30–40% of the head cells. As *fumble* is likely expressed in all cells, this suggests that the *fumble* is almost completely silenced in the *tim-*expressing cells. In addition, the one predicted off-target of the used RNAi transgene (*CG15923*) is not changed in the heads of tim-fbl flies relative to controls (the gene is not expressed, with a log 2 intensity value <4).

**Figure 5:**
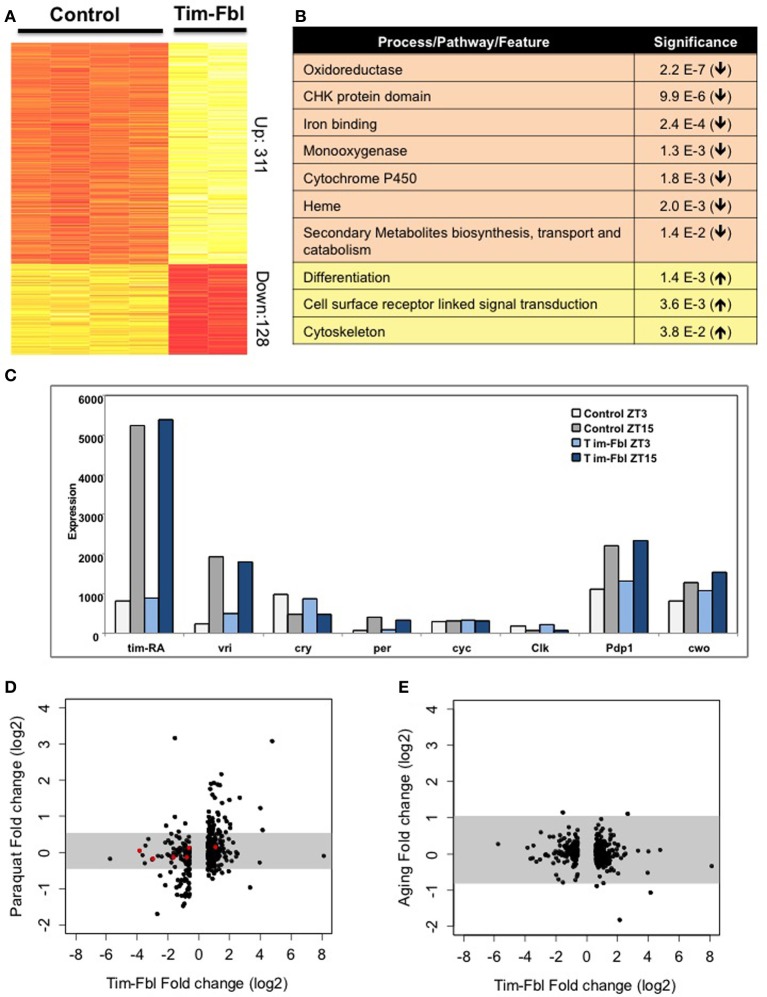
**The tim-fbl flies display a specific transcriptional signature. (A)** Genes differentially expressed in heads of tim-fbl females compared to control flies. 443 probes were up or down regulated by over 1.5 fold. Yellow indicates upregulated and red downregulated. **(B)** DAVID analysis showed pathways enriched in genes with altered expression. **(C)** Tim-Fbl female flies have normal levels of core circadian components in light:dark cycles. **(D)** Changes in gene expression following paraquat treatment compared with the changes observed in fbl mutants. Each point is the fold change for one of the 443 genes showing significant differential expression with FDR < 0.15, and fold change above 1.5 in the tim-fbl experiment. The *Y* and *X* axes are on a log scale, base 2. The gray horizontal bar is the interval that contains 99% of the fold changes measurements in the paraquat (left) or aging (right) experiments. **(E)** Changes in expression with aging as a function of the changes in the tim-fbl experiment. The plot was performed as in **(C)**.

We performed an exhaustive statistical analysis, and selected genes with a fold change (in either direction) of more than 1.5 fold and an FDR *q*-value < 0.05. These parameters retrieved 31 probes. In order to establish pathways and specific genes affected in tim-fbl flies, we utilized less stringent threshold (FCR < 0.15). Because of the spatially restricted nature of the tim-gal4 driver, a change in expression of 1.5 fold in whole-head RNA suggests an approximate 2–3 fold change in the target cells. We found that 317 genes were significantly upregulated and 126 were downregulated in tim-fbl flies (Figure 5A; Supplementary Files 1 and 2). Interestingly, and in concordance with our luciferase results (Figure 3A), the oscillation and levels of core circadian components was not affected in tim-fbl flies (Figure 5C).

We determined the processes and/or pathways affected by the genes with altered expression in tim-fbl flies compared to controls. Although some specific stress genes were highly upregulated in tim-fbl flies (e.g., *AttD, LysX*, and genes that express several Mth-like proteins), genes involved in stress or detoxification pathways were not significantly upregulated in these flies. Interestingly, differentiation, cell surface receptor linked signal transduction, and cytoskeleton genes were enriched among the upregulated genes in tim-fbl flies (Figure 5B). Indeed, cytoskeleton defects (in the form of acetylation of tubulin) have been recently demonstrated to be a downstream effect of *PanK* deficiency in flies and mammalian cells (Siudeja et al., 2011). Importantly, genes involved in specific mitochondrial pathways were significantly downregulated in these young tim-fbl flies, including those encoding heme-binding proteins and cytochrome P450 (Figure 5B).

PKAN has been associated with the production of oxidative stress species, yet as expression data from PKAN patients or animal models is lacking, it is not known whether *PanK* deficiency leads to a transcriptome signature of oxidative stress. We compared the gene profile of tim-fbl flies to those of flies exposed to paraquat by incorporating publicly available transcriptome data from heads of control flies and flies exposed to 5 mM paraquat (GEO accession number GSE35930). There was significant overlap between the genes upregulated or downregulated upon paraquat exposure with those affected in tim-fbl flies (Figure 5D). This was not a consequence of the statistical parameters we chose. Indeed, utilizing the same approach with expression data on aged flies (Wood et al., 2010), we did not find any significant similarity between genes changed in tim-fbl flies and genes differentially expressed in older flies (Figure 5E). In any case, tim-fbl flies also displayed oxidative-stress-independent signatures including those of genes related to the eye pigment biosynthetic pathway (Figure 5D, red dots).

**Figure 6:**
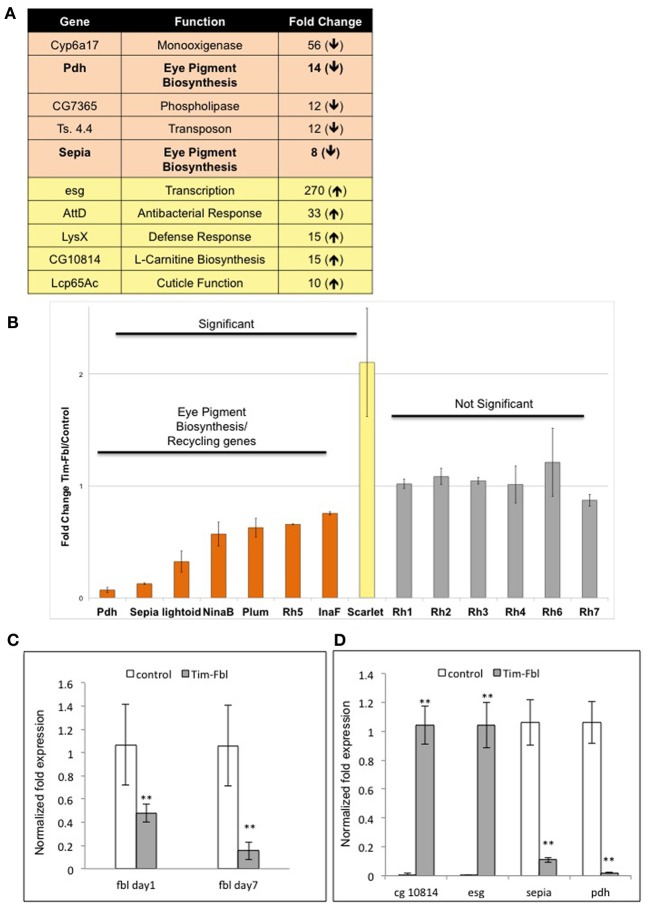
**Microarray data point to eye degeneration as a highly altered pathway in tim-fbl flies and validation of microarray expression analysis by Real-time PCR. (A)** A number of genes with altered expression in tim-fbl flies are reported to play a role in eye degeneration. **(B)** Eye Pigment Biosynthesis and Pigment Recycling genes are significantly changed in tim-fbl flies, mostly downregulated. In contrast structural eye genes are not significantly altered. Error bars denote standard deviation. The statistical significance is based in FDR assessments as in Figure 4. (**C** and **D**) Expression levels of selected genes were identified by using real-time PCR. Data was normalized to the expression of the housekeeping genes tubulin and rp49. Error bars denote standard error of the mean (*n* = 3, ^**^p > 0.001). In both cases tim-fbl females were raised at 18°C and upon eclosion where transferred to 25 and collected after one or seven days. In **(C)**, flies were collected after seven days at 25°C.

Among the 10 genes most affected in tim-fbl flies, we surprisingly found two related to eye pigment biosynthesis (Figure 6A, *pdh*, number 3 and *sepia* number 8). These two genes are downregulated 14- and 8-fold, respectively, and are not among the oxidative stress signature genes. As stated above mutations in the *pdh* gene provoke light-dependent loss of rhodopsin and retinal degeneration. In addition to *pdh* and *sepia*, other genes related to eye pigment biogenesis (and hence maybe recycling) are significantly downregulated in tim-fbl flies: *lightoid, ninaB*, and *Plum* (3-, 1.6-, and 1.7-fold decreases relative to control, respectively); and one (*scarlet*) is 2-fold upregulated (Figure 6B). Levels of these genes are not changed in a similar fly neurodegenerative model [polyglutamine-expanded protein under the control of tim-gal4 driver (Kadener et al., 2006)] or upon oxidative stress induced by paraquat (Figure 5D). Therefore, this downregulation constitutes a landmark of *dPanK* deficiency and is not a downstream effect of toxicity in photoreceptors. Supporting this hypothesis, the levels of most of the structural eye genes (encoding rhodopsins 1, 2, 3, 4, 6, and 7) are unaffected in tim-fbl flies and no external eye defects were observed (Figure 6B). This suggests that the change in the transcriptional program in tim-fbl flies, rather than the chronic exposure to oxidative stress, may lead to the retinal degeneration widely observed in *dPanK*-deficiency models. In order to validate the microarray results in our milder PKAN “inducible” model, we performed RT-PCR in tim-fbl flies grew at 18°C and transferred them to 25°C for 7 days. Indeed *fbl* was downregulated in those flies even after only 1 day at 25°C (Figure 6C). Moreover the four other genes tested by RT-PCR showed a similar expression profile than in the microarray assay (*sepia* and *pdh* downregualted in tim-fbl flies and *esg* and CG10814 upregulated in the PKAN model; Figure 6D).

**Figure 7:**
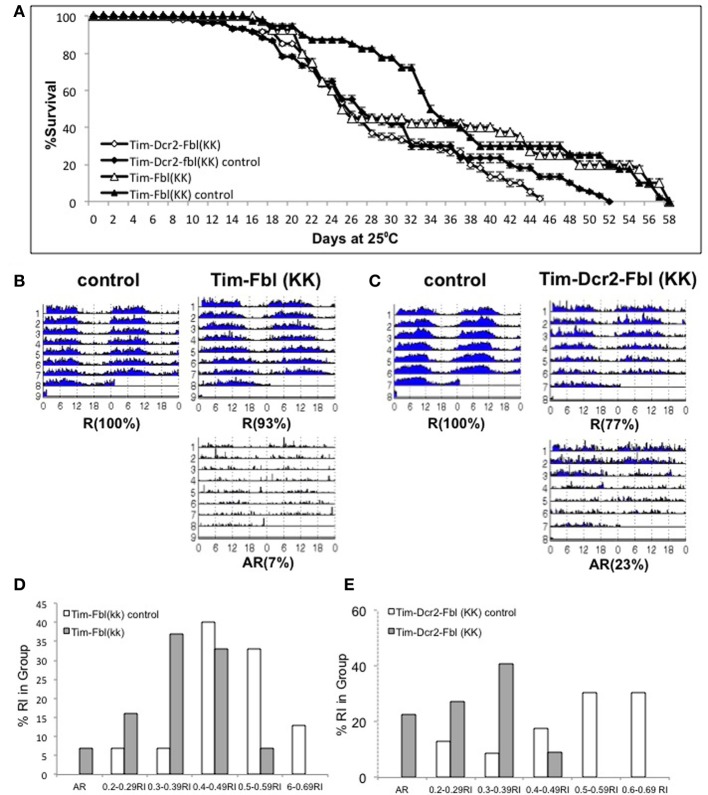
**Validation of the PKAN model using a second UAS-fbl RNAi transgene. (A)** Percent survival curve of tim-fbl and tim-dcr2-fbl (KK) flies. Both males and females (tim-fbl and tim-dcr2-fbl KK flies) flies raised at 25°C. We utilized sibling flies from the cross as controls (20 flies per vial, *n* = 3). **(B)** Circadian locomotor behavior of tim-fbl (KK) flies is characterized by reduced rhythmicity in respect to controls. Actograms of female tim-fbl (KK) along with their respective controls (sibling flies) represent activity in constant conditions immediately after 3 days of 12:12 light:dark entrainment and show reduced rhythmicity of tim-flb (KK) flies. **(C)** Similar than in **(B)**, but for tim-dcr2-fbl (KK) flies. **(D)** Tim-Fbl flies have weaker rhythms than control flies. Histogram representing relative frequency of flies at different rhythm indexes (RI) intervals. The graph was constructed as indicated for Figure 4F. **(E)** Similar to **D** but for Tim-dcr2-fbl (KK) flies (*n* = 64).

**Figure 8:**
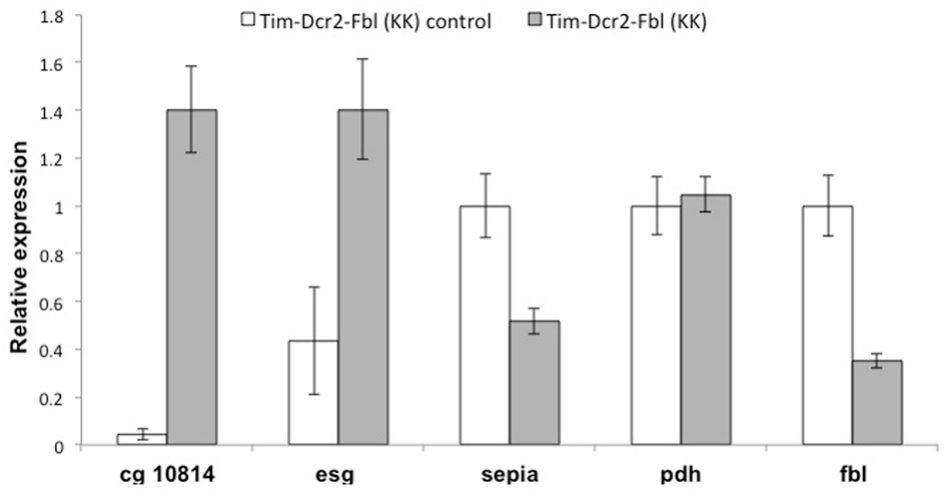
**Gene expression profile of tim-Dcr2-fbl (KK) flies resembles the effects observed one in tim-Fbl flies.** Tim-Dcr2-fbl (KK) flies raised at 25°C, were maintained in 12:12 light:dark cycles for seven days after eclosion and fly heads collected at ZT3. We extracted RNA from these fly heads and use it to perform RT-PCR against five genes with significant gene expression differences in Tim-Fbl flies (respect to control flies, see text). Four out of those five genes were affected in the same direction in tim-dcr2-fbl (KK) flies. Data was normalized to the expression of the housekeeping genes tubulin and rp49, (*n* = 3). Error bars denote standard error of the mean.

**Figure 9:**
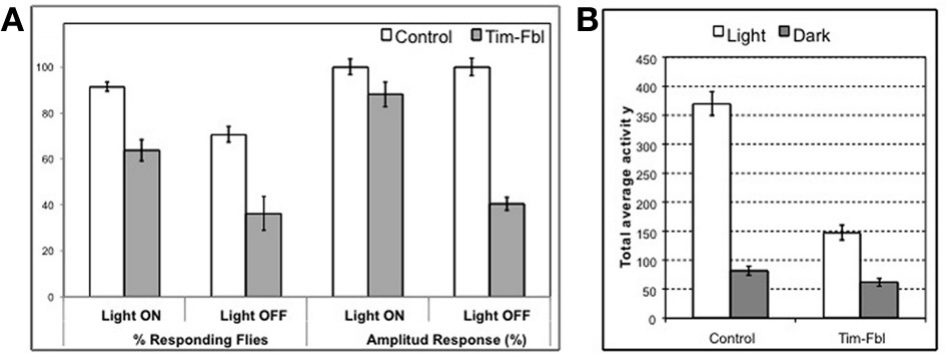
**Behavioral assays indicate impaired vision in tim-fbl flies. (A)** The tim-fbl flies have reduced responses to light-dark transitions, an expected functional outcome of impaired vision. Number of responding flies and activity response amplitude are both strongly decreased in tim-fbl flies, both in light-on and light-off events. Response to both events is independent of the circadian clock and in the case of lights-on is dependent on functional vision. **(B)** Total locomotor activity is reduced in tim-fbl flies. The total activity of tim-fbl flies is diminished, and this reduction is more prominent in daytime activity (*n* = 32).

One common problem while using RNAi transgenes is off-targeting (Seinen et al., 2010, 2011). In order to confirm that the observed phenotypes are due to downregulating of *fumble* and not to secondary effects of the specific RNAi transgene, we tested a different UAS-RNAi transgene against *fbl*. We utilized a transgene from the KK collection (VDRC, Vienna), which is part of an independent RNAi library (different region in *fbl* gene and different insertion site). We expressed this transgene using the tim-gal4 driver with or without a UAS-dicer2 transgene [Tim-fbl(KK) or Tim-dcr2-fbl (KK) flies]. Interestingly, Tim-fbl (KK) and Tim-dcr2-fbl (KK) flies displayed more subtle phenotypes regarding survival than tim-fbl flies (Figure 7A). However, both strains showed significant circadian behavioral phenotypes (Figures 7B,C). These phenotypes are more severe in tim-dcr2-fbl flies (more percentage of arrhythmic flies and weaker rhythms of rhythmic flies; Figures 7D,E). The somehow milder phenotypes observed with this RNAi second transgene flies are likely related to lesser efficient silencing of *fbl*, as determined by RT-PCR (compare *fbl* in Figure 9 with Figure 6C at day 7). In addition to behavioral defects, tim-dcr2-fbl (KK) flies have changes in gene expression that strongly resemble the ones observed in Tim-fbl flies (four out of five genes are affected in a similar way in tim-dcr2-fbl (KK) flies; Figure 8, compare with Figures 6C,D). This demonstrates that both the behavioral as well as the transcriptome results described for Tim-fbl flies are consequence of *fumble* downregulation and not related to the genetic background or off target effects of the RNAi transgene.

### Tim-fbl flies display impaired light behavioral responses

As described above, we observed a significant downregulation of the eye-pigmenting pathway enzymes in tim-fbl flies. In order to test whether this downregulation leads to eye functional defects, we measured the capacity of tim-fbl flies to detect light-dark transitions. We grew tim-fbl flies at 18°C, transferred them to 25°C upon eclosion and maintained them in presence of light:dark cycles for 1 week. In presence of light, chromosphere recycling is essential for maintenance of retinal integrity (Wang et al., 2010). In these conditions and over this time frame, tim-fbl flies do not die and hence functional photoreceptor-specific defects can be assessed. Flies exposed to light:dark cycles responded acutely to lights-on and lights-off events with an immediate boost of locomotor activity over a timescale of minutes. At least in the lights-on case this response is dependent on a functional eye (Rieger et al., 2003). We focused on the last four days of the assay and measured and compared the locomotor activity of control and tim-fbl flies in the 10 min preceding the transition with the 10 min after the transition. As shown in Figure 9A, control flies responded strongly to light transitions, with more than 90% of the flies responding to the lights-on event and almost 80% of the flies responding to the lights-off effect. However, the response of tim-fbl flies was weaker with 60% of the flies responding to the lights-on and less than 40% to the light-off signal (Figure 9A, % responding flies). In addition, the amplitude of the response was severely diminished for the lights-off event (Figure 9A, right columns).

*Drosophila melanogaster* is a diurnal insect, and the compound eye controls this diurnal pattern of activity. For example, in our hands control flies are 4.5 times more active during the light period (Figure 9B); tim-fbl flies were significantly less active during the light period (by ~50%, Figure 9B). However, this difference was mainly due to lower activity during the day. Indeed, tim-fbl flies showed only a 2-fold difference in the activity levels between the light and dark period providing further evidence that these flies have vision defects. These data constitute strong proof that the eye visual pathways are affected in tim-fbl flies.

## Discussion

In the present study we developed a new model for the neurodegenerative disease PKAN in *Drosophila*. We did so by restricting the disease to the cells harboring a circadian clock. Using this new type of approach we can follow the progression of the disease in real-time in live flies by monitoring physiological and behavioral outputs of the circadian system. We generated both constitutive and inducible models of PKAN. Both of these models recapitulate essential aspects of *PanK*-deficiency including shortened life span, hypersensitivity to oxidative stress, and brain-specific behavioral defects. More specifically, tim-fbl flies had aberrant circadian locomotor rhythms, which are likely due to neuronal defects. Moreover, we demonstrated that tim-fbl flies display a specific transcriptional signature that may underlie the prevalent retinal degeneration observed in murine and fly PKAN models and PKAN patients. This unique profile was accompanied by impaired light-mediated behaviors that preceded development of visible eye phenotypes. Using this model we found that the transcriptional response to CoA deficiency may have a key and previously unsuspected role in this devastating disease.

Onset of PKAN, like that of other neurodegeneration with brain iron accumulation (NBIA) disorders, occurs at early age (Schneider and Bhatia, 2012). The symptoms of PKAN progressively worsen, and most symptoms, although not all, relate to muscle incapability, with retinal degeneration as landmarks of the disease (Schneider and Bhatia, 2012). Prior to our work, two genetic animal models of PKAN had been created. Although the use of the mouse *PanK* knockout model is extremely limited [due to the mild phenotype, (Kuo et al., 2005)], the *Drosophila* model (*fumble* mutant) has been extremely useful (Yang et al., 2005; Bosveld et al., 2008; Wu et al., 2009; Rana et al., 2010). However, the *fumble* mutant *Drosophila* model has many technical disadvantages. Among them is high developemental lethality [almost 100% (Afshar et al., 2001)], sterility, and general sickness of *fumble* flies that precludes their use in many biochemical assays and in screens for modifiers of toxicity. In addition, the multiple and severe phenotypes observed in these flies makes it difficult to distinguish between early and late pathological events or between general and neurological (brain-specific) events.

Our model offers a solution to these previous shortcomings. The tim-fbl flies display general phenotypes that resemble those of PKAN patients and *fumble* flies such as diminished lifetime, hypersensitity to oxidative stress, and diminished activity. However, these phenotypes are significantly less severe than in the other fly model and tim-fbl flies are fertile (at least the ones grew at 18°C), which will allow their use in future genetic screenings. These differences may be due to a different degree of reduction in *fumble* expression between cells of the two models or to the tissue-restricted repression in tim-fbl flies. Although we favor the later hypothesis, the tim-gal4 driver is broad enough to generate general effects on viability and life span in tim-fbl flies. Despite these general effects, tim-fbl flies display specific behavioral phenotypes that are associated with the restrictive expression of the tim-gal4 transgene in the brain. For example, tim-fbl flies show climbing abilities at least at young age (Figure 4H) identical to those of control flies, even though they display aberrant circadian rhythms (Figures 4A,B). Although we use the circadian cells in the fly brain as a cellular model for understanding the consequences of PANK deficiency, the circadian system may be a relevant system to look in relationship to PKAN. Recently, it was reported that PKAN patients display abnormal circadian patterns of sleep-wake cycles as the result of their altered sleep architecture (Fantini et al., 2010). More specifically, Fantini et al. found that PKAN patients have less sleep hour and more REM-sleep related behavior disorders than control cases (Fantini et al., 2010). Despite this connection, we acknowledge that other neuronal groups may be more relevant even in the fly model. In this context it would be interesting to compare the behavioral effects that are obtained after knocking down *dPANK* in different neuronal groups in *drosophila*.

Importantly, the behavioral circadian defects observed in tim-fbl flies likely reflect neuronal defects (i.e., output) rather than general health impairment of the circadian molecular cycle. This may be surprising given the aberrant behavioral rhythms displayed by these flies (Figure 4A). In order to distinguish between direct transcriptional effects due to the *fumble* downregulation and those that are a consequence of circadian defects, we evaluated gene expression by microarray analysis at two different time points in tim-fbl and control flies. After exhaustive statistical analysis, we did not find changes in the levels of circadian components (e.g., *tim, per, vri*) nor downstream effectors of the circadian clock, demonstrating that the general circadian transcriptional machinery is unaffected in tim-fbl flies (Figure 5C). This is in sharp contrast to the effect that expression of proteins with polyglutamine expansions provoke in the oscillations of core clock components (Kadener et al., 2006). Hence, we favor the notion that the behavioral defects of tim-fbl flies arise from circadian neuronal network defects rather than from general cell-burden or impairment of the transcriptional machinery. Indeed, the fact that tim-dcr2-fbl (KK) flies show altered circadian rhythms without major effects on lifespan strongly argues in this direction. The neuronal defects may be due to neuronal transmission defects consequence of the altered cytoskeletal properties produced by CoA deficiency, as recently noted in *fumble* mutant flies (Siudeja et al., 2011). The luciferase-based reporter experiment also supports this hypothesis: Although total levels of luciferase are diminished, this decrease is about 25–30%, and we did not observe effects in the timing or amplitude of the transcriptional circadian cycle.

The reduced luciferase levels could also be a consequence of a more general defect. For example, reduced general transcription and protein translation and lower ATP levels may all lead to this phenotype, as the transgenic luciferase uses ATP for luminescence and is degraded rapidly, rendering luminescence dependent on translation, and possibly transcription. CoA itself may play a direct role in luciferase luminescence complicating comparison to other fly lines without CoA-relevance. Indeed, reduced transcription and translation and lowered-ATP-levels may all derive from CoA insufficiency, the most probable initial lesion in PKAN and *fumble* (Rana et al., 2010).

Our approach allowed us to generate a tunable model in which disease can be aggravated during adulthood (Figure 1B). Although tim-fbl flies grown at 18°C displayed almost no embryonic lethality, after transfer to 25°C they displayed a short life span and hypersensitivity to oxidative stress due to expression of the UAS-RNAi transgene (Figures 1B, 2B). This model will provide the unique opportunity for genome-wide genetic screenings for modifiers (suppressors or enhancers) of *PanK*-deficiency toxicity.

**Figure 10:**
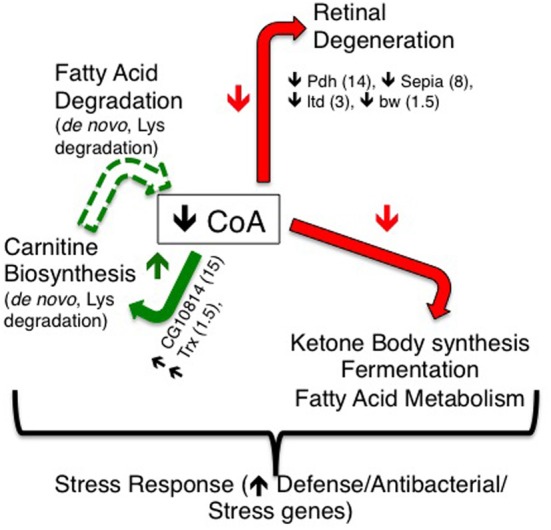
**A model for resulting transcriptional effects of *dPanK* downregulation in tim-fbl flies.** *dPanK* downregulation is known to lead to reduced CoA biosynthesis, which may explain the transcriptional changes observed in this study. In tim-fbl flies, *dPanK* downregulation results in a marked reduction in several genes previously implicated in retinal degeneration (including *Pdh, Sepia, ltd*, and *bw*). Additionally, several genes involved in carnitine biosynthesis (including the genes *CG10814* and *Trx*) are upregulated, which may feed forward to reduced CoA biosynthesis by altering fatty acid degradation. These, together with ketone body synthesis, and fermentation may represent key transcriptional effects indicative and possibly causative of PKAN.

In order to identify factors and pathways involved in *PanK*-deficiency pathogenesis, we performed gene expression analysis of control and tim-fbl female flies. We found that tim-fbl flies have a unique transcriptional signature compared to control flies (see scheme in Figure 10). Among the upregulated genes were those involved in cytoskeletal function. Indeed cytoskeleton defects have been observed in *fumble* flies and Pank deficient mammalian cells (Siudeja et al., 2011). Certain genes are likely upregulated in an immediate response to the drop of CoA levels upon inhibition of *dPanK* expression. For example, CG10814, an enzyme with gamma-butyrobetaine dioxygenase activity involved in the synthesis of carnitine [the main transporter of acetyl-CoA into the mitochondria (McGarry and Brown, 1997)] was 15-fold upregulated. Many stress response genes were also upregulated in the tim-fbl flies, although to a much lower degree than in other mitochondrial/neurodegenerative disorder models. This may be due to the fact that we performed the gene expression analysis in young flies.

There is also a clear shutdown in metabolic processes in the tim-fbl flies. We speculate that this is consequence more of a “cellular decision” in order to avoid oxidative damage rather than mitochondrial death or impairment. This is based in the fact that only specific pathways are downregulated and that some mRNAs in these same pathways are even upregulated, suggesting that this unique gene expression profile could be due to “uncoordination” due to the low CoA levels, an atypical physiological situation. Among the pathways that are downregulated in tim-fbl flies are those of genes involved in pigment biogenesis. It has been recently shown that the product of one of these genes, *pdh*, is required for recycling of the chromophores in the eye and that lack of this enzyme leads to retinal degeneration (Wang et al., 2010). *Pdh* is downregulated 15-fold in tim-fbl flies (Figure 6A; Supplementary File 1). In addition at least four other mRNAs involved in this pathway are significantly downregulated (Figure 6B).

The current hypothesis is that the retinal degeneration phenotype is at least partially due to oxidative stress, a downstream effect of CoA deficiency and likely parallel to the brain neurodegeneration phenotype (Kuo et al., 2005). However, our data suggests that at least in our model the role of oxidative stress is more limited. First, although the tim-fbl fly transcriptome strongly resembles that of flies subjected to oxidative stress through paraquat exposure, the chromophore-recycling pathway constitutes a clear exception, meaning that oxidative stress alone is not enough to deactivate this pathway. Second, other neurodegenerative diseases linked to oxidative stress (i.e., polyglutamine disease) do not show changes in the expression of the enzymes of this pathway (Kadener et al., 2006). Third, tim-fbl flies show an impaired behavioral response to light (although not a complete loss) before they show any signs of diminished health or visible eye morphological defects. All the above strongly suggest that retinal degeneration in fly models of PKAN (ours and *fbl* flies) and even in patients may be due to a change in the transcriptional profile rather than consequence of oxidative stress. In this context, it would be interesting to test whether the defects in tim-fbl flies are rescued by overexpression of the downregulated genes. However, such an experiment requires an immense number of transgenes and hence may result in non-specific phenotypes.

A significant number of tim-fbl flies respond to the lights-on event (which is entirely eye-dependent) suggesting that at least at the age that the flies were assayed, the eye degeneration is ongoing (Rieger et al., 2003). The tim-fbl flies do not display an obvious eye phenotype, and the levels of most rhodopsin mRNAs are not affected. Hence, our results strongly suggest that the retinal degeneration phenotype widely observed in PKAN patients and animal models (mouse and *Drosophila*) is consequence of a specific transcriptional signature in CoA-deficient situation, rather than a result of oxidative stress. It is important to point out that we performed these photoresponse experiments with tim-fbl flies grown at 18°C, so these defects are likely not developmental.

In sum, in the present study, we demonstrated an unexpected transcriptional signature to *dPanK* deficiency suggesting that these defects may be a key aspect of PKAN. Moreover, in the present study we developed a new type of neurodegeneration fly model that can be applied to other diseases and that allows the assessment of neurodegeneration in the brains of live flies.

## Materials and methods

### Fly stocks and maintenance

Flies were reared at 25°C on a standard diet {yeast: 38 gr/L, Yellow corn mill: 91 gr/L, agar: 10 gr/L, molassas: 8.7% v/v, propanoic acid (BioLab): 0.9% v/v, Tegasept solution [Sigma-Aldrich; 300 g/L in EtOH (BioLab)]: 0.8% v/v}. Flies were kept in 12-h light:12-h dark cycles at 25°C, except during experimental manipulation Transgenic fly lines used in this study were originally described: tim-Luc flies (Allada et al., 2003), *tim-*GAL4 (FBti0017922; Kaneko and Hall, 2000)UAS-*dcr2* (FBrf0200691; Dietzl et al., 2007) UAS RNAi lines against *fbl* obtained from the Vienna *Drosophila* RNAi Center (Dietzl et al., 2007); VDRC- 44157 and 101437 (KK). Stable recombined strains of the above *tim-*GAL4 and tim-Luc lines were used in real-time luciferase detection.

### Developmental lethality

To assess the developmental lethality, we crossed UAS-fbl IR male flies with tim-gal4 virgin females flies at 25°C in bottles and supplemented with standard fly food. The bottles were kept in 12:12h light:dark regime with controlled humidity. After three days flies were passed into new bottles and emerged flies were counted upon eclosion. The percentage was calculated relative to the expected appearance of the phenoytpe.

### Death curves

Crosses of both tim-gal4 and tim-gal4-dcr2 flies with UAS-fbl IR were carried out in either 18 or 25°C. Three days old progeny, both males and females and their respective sibling controls were divided to duplicates plastic vials and counted every one or two days. The tubes were kept laying in 12-h light:12-h dark cycles at 25°C, and changed every 2–3 days. Experiments were repeated out no less than three times.

### Paraquat sensitivity assay

Twenty flies (3 days old Male and female) of tim-fbl and respective sibling controls in duplicates where feed with standard food or food containing 1mM or 3mM paraquat (1,1-dimethyl-4,4-bipyridinium dichloride, Sigma-Aldrich). Growth conditions, passing to new vials and counting was performed as reported for death curves. Experiments were carried out no less than three times.

### Real-time luciferase detection

Bioluminescence measurements of individual live flies were performed using a Packard Topcount NXT machine. 96-well plates (Optiplate; Packard) were filled with 100 μl Luciferine-contating food consisting of 1% agar, 5% sucrose, and 25 mM Luciferin (Biosynth) dissolved in H_2_O. Flies were entrained in plates for at least 2 days in LD, followed by 5–7 days in constant darkness (DD). Luminescence was measured Approx. once per hour. Only reads of flies which lived thought the assay were used, and reads normalized similarly to eliminate linear decay of emission.

### Locomotor behavior

Adult females flies (3–7 days old) were placed in glass tubes and monitored for 2–3 days in LD, followed by 7–14 days in DD, using the *Trikinetics Drosophila Activity Monitors* (DAM; Trikinetics, Waltham, MA) system. During assay flies were fed by sucrose-containing agar (2% Agar, BD; 5% sucrose, BioLab), and maintained in fixed humidity and temperature (25 ± 1°C OR 29 ± 1°C) conditions. The number of beam-breaks occurring in 5-min time-bins was obtained by DAM System 3 Data collection software, and data analyzed using MatLab, as described (Levine et al., 2002). Rhythm index of >0.2 in DD was used as threshold for defining activity as rhythmic.

### Activity and light response behavior

Flies were kept and monitored as reported for assaying locomotor activity. The number of beam-breaks occurring in 1-min intervals was recorded. In order to calculate the acute behavioral response to light-on or off events, we calculated the total activity events in the 10 min preceding the light transition even and compared with the activity after the transition. We consider a positive response if the differential is more than 2 activity counts.

### Climbing assay

Climbing assay was adopted from (Bosveld et al., 2008) with numerous changes, as all flies are absolute climbers in our model system. Three day-old flies were anesthetized by CO_2_ and pre-grouped in vials. After >20 min flies were inserted/poured from vials without anesthetization to a graduated cylinder. Flies were gently tapped to bottom of cylinder and allowed to climb for 15 s. Climbing of individual flies was scored in a gradual manner by the height they reached, and score averaged over group. Five climbing sessions were carried out for each of 3 repetitions/groups of ~20 same-age flies, both males and females, for each genotype.

### Microarray assessment and analysis

New born control or tim-fbl female flies were entrained for three days in 12:12 light:dark conditions at 25°C and collected at ZT3 or 15 (three hours after the light on or lights off event). RNA was extracted using the Zymo RNA extraction kit. RNA was then used to prepare a probe that was hybridized to *Drosophila* 2.0 gene expression arrays (Affymetrix). CEL files were imported to Affymetrix Expression Console program, and were normalized using RMA (Robust Multichips Analysis). Statistical analysis was performed using the R statistical environment (http://cran.r-project.org/). Differential expression was tested for all genes that had expression levels above 4.0 (log2 scale) for all measurements in at least one group (mutants or WT). To test for differential expression we used analysis of variance (ANOVA), controlling for the effect of time of day. The *p*-values from the ANOVA were adjusted for multiple testing using the false discovery rate (FDR) correction implemented in the R-function p.adjust.

### Analysis of gene expression by real-time PCR

Total RNA was extracted by using Trizol reagent (*Invitrogen*) from 7 day old control and tim-fbl or tim-dcr2-fbl fly head, which were kept at 12:12 LD condition at 25°C and collected at ZT3. cDNA synthesis was carried out as described in the BIO-RAD iSCRIPT™ cDNA synthesis kit and Dnase treatment given by promega kit. Quantitative Real time PCR was performed by using cDNA as templet derived from this RNA with BIO-RAD (C 1000™ Thermal cycler) real time pcr. The PCR mixture contained Platinum Taq polymerase (Life Technologies), optimized concentrations of Sybrgreen and the following primers: *sepia*: 5′-TATGATTTGGCCCTGGTGT-3′, 5′-CCATCACAGCCGGATCTC-3′, *pdh*: 5′-ATGCCCGAGCGTTGATAGTA-3′, 5′-TGGCATTGTGGTTAACATGAG-3′, *fbl*: 5′-TTACAACCGCTTTGGTCTCC-3′, 5′-CCACAAAGACGACCTATCG-3′, *cg10814*: 5′-GGAATGCTGTGGTTGATG-3′, 5′-GAGCAGTTCCGGGTTCTTT-3′, *esg*: 5′TTCCACATGTCGCCCTACAC-3′, 5′-AATAAGCCGGCGAGATAGGC-3′, *rp49*: 5′-ATCCGCCCAGCATACAG-3′, 5′-TCCGACCAGGTTACAAGAA-3′

Cycling parameters were 95°C for 3 min, followed by 39 cycles of 95°C for 10 s, 55°C for 10 s, and 72°C for 30 s. Fluorescence intensities were plotted vs. the number of cycles by using an algorithm provided by the manufacturer. mRNA values from heads were normalized to that from housekeeping gene tubulin and ribosomal protein 49 (*rp49*).

### Conflict of interest statement

The authors declare that the research was conducted in the absence of any commercial or financial relationships that could be construed as a potential conflict of interest.

## Supplementary material

Supplementary File 1 **Genes upregulated in tim-fbl flies**.

Supplementary File 2 **Genes downregulated in tim-fbl flies**.
