# Erratum: A new *in vivo* model of pantothenate kinase-associated neurodegeneration reveals a surprising role for transcriptional regulation in pathogenesis

**DOI:** 10.3389/fncel.2013.00187

**Published:** 2013-10-23

**Authors:** Frontiers in Neuroscience Editorial Office

**Keywords:** NBIA, PKAN, PanK, circadian rhythms, Coenzyme A, Drosophilia

In the authors list the given names and surnames were inverted. The authors list should have been: Varun Pandey, Hagit Turm, Uriya Bekenstein, Sagiv Shifman and Sebastian Kadener.

